# Physicochemical and Adsorption Characterization of Char Derived from Resorcinol–Formaldehyde Resin Modified with Metal Oxide/Silica Nanocomposites

**DOI:** 10.3390/ma17091981

**Published:** 2024-04-24

**Authors:** Mariia Galaburda, Dariusz Sternik, Agnieszka Chrzanowska, Olena Oranska, Yurii Kovalov, Anna Derylo-Marczewska

**Affiliations:** 1 Faculty of Chemistry, Maria Curie-Skłodowska University, Maria Curie-Sklodowska Sq. 3, 20-031 Lublin, Poland; dariusz.sternik@mail.umcs.pl (D.S.); agnieszka.chrzanowska@mail.umcs.pl (A.C.); 2 Chuiko Institute of Surface Chemistry, National Academy of Sciences of Ukraine, 17 General Naumov Str., 03164 Kyiv, Ukraine; el.oranska@gmail.com; 3 School of Chemistry, University of Bristol Cantock’s Close, Bristol BS8 1TS, UK; ununulium123@gmail.com

**Keywords:** metal oxide, resorcinol–formaldehyde resins, silica, pyrolysis, carbon composites, char, adsorption of organics

## Abstract

A series of metal- and silica-containing carbon-based nanocomposites were synthesized by pyrolysis of a resorcinol–formaldehyde polymer modified with metal oxide/silica nanocomposites (MxOy/SiO_2_, where M = Mg, Mn, Ni, Cu and Zn) via the thermal oxidative destruction of metal acetates adsorbed on highly dispersed silica (A380). The concentration of metals was 3.0 mmol/g SiO_2_. The phase composition and morphological, structural and textural properties of the carbon materials were analyzed by X-ray diffraction, SEM, Raman spectroscopy and low-temperature N_2_ adsorption. Thermal decomposition under a nitrogen atmosphere and in air was analyzed using TG–FTIR and TG–DTG–DSC techniques to determine the influence of the filler on the decomposition process. The synthesized composites show mesoporous structures with high porosity and narrow pore size distributions. It could be shown that the textural properties and the final composition of the nanocomposites depend on the metal oxide fillers of the precursors. The data obtained show that nickel and copper promote the degree of graphitization and a structural order with the highest porosity and largest specific surface area of the hybrid composites. The good adsorption properties of the obtained materials were shown for the recovery of p-chlorophenol and p-nitrophenol from aqueous solutions.

## 1. Introduction

Silica and carbon play an important role in materials science due to their excellent physical and chemical properties. Nanomaterials based on carbon and silica have several advantages, such as a large surface area, robust structural stability, diverse shapes, tunable structures and compatibility with biological systems [1,2,3]. Such hybrids can potentially combine the advantages of the individual materials to create a composite material with improved surface properties, adsorption capabilities, chemical and hydrothermal stability and high conductivity so that they can be used, for example, as catalyst carriers, in the energy sector, as thermal insulators, in separation processes, as adsorbents for large and small organic molecules and heavy metal ions, etc. [4,5,6,7,8,9].

The porous carbonaceous materials can be obtained by various physicochemical methods based on different raw materials, coals and polymers using combinations of templating and carbonization processes [10,11,12,13,14,15]. The properties of carbon nanocomposites, such as porosity and structure, are determined by a variety of factors, including synthesis methods, precursor materials and processing conditions. Resorcinol–formaldehyde resins are particularly notable for their ability to produce highly porous carbon materials when used as precursors [16,17]. The polymerization reaction between resorcinol (R) and formaldehyde (F) is a spontaneous and slow process. To increase the reaction rate and shorten the synthesis time, basic or acidic compounds are often used, which significantly influence the reaction mechanisms and the final structure as well as porosity and morphology of the resulting carbon materials [18,19,20]. Various metals, such as Fe, Co, Ni, Pd, Ag and their respective species, have been used for the synthesis of RF-based polymers and their carbon-based composites [21,22,23,24]. The main disadvantage of using salts in the co-condensation reaction of R and F is the uneven distribution of the metal during the gelation process and thus in the carbon during pyrolysis. The process of impregnation with fumed silica led to a uniform distribution of the metal filler over the matrix surface, resulting in a homogeneous coverage of the silica surface [25].

Polymer-derived carbon composite materials are used in various fields, including electrochemistry (energy storage, supercapacitors and fuel cells), catalysis, adsorption, etc. [26,27,28,29]. Such composites are effective materials for sorption processes or for the preliminary separation and concentration of analytes in analytical procedures (solid-phase extraction (SPE) technique). For example, Tomaszewski et al. obtained promising results in applying the SPE technique for the extraction of explosive nitramines from post-blast soil samples using similar systems containing carbosil. It was found that the recovery of explosives on carbosil can be controlled by the amount of carbon deposition and surface area accessible to the analytes, while additional silylation of the materials can alter their adsorption [30]. Therefore, it is of interest to investigate the adsorption characteristics of the composite materials of divergent texture and surface properties with regard to the recovery/extraction of selected organic substances from liquid media to evaluate the influence of adsorbent properties on the performance of adsorption/extraction processes.

The aim of this study was to investigate the influence of metal oxides on the textural, structural and adsorption properties of the silica-containing carbon composites C/Me/SiO_2_, where Me is Cu, Mg, Mn, Ni and Zn. Besides the physicochemical characterization, the adsorption properties were also analyzed for the recovery of 4-nitro- and 4-chlorophenol from aqueous solutions, and the effect of composite characteristics on sorption effectiveness was analyzed.

## 2. Materials and Methods

### 2.1. Chemicals

Aerosil A-380 (Evonik Degussa GmbH, Essen, Germany), magnesium acetate tetrahydrate, Mg(CH_3_COO)_2_·4H_2_O (ACS reagent, ≥98%, Merck, KGaA, Darmstadt, Germany), manganese(II) acetate tetrahydrate, Mn(CH_3_COO)_2_·4H_2_O, (99.99%, Sigma-Aldrich, KGaA, Darmstadt, Germany), nickel(II) acetate tetrahydrate, Ni(CH_3_COO)_2_·4H_2_O (98%, Sigma-Aldrich), copper(II) acetate hydrate, Cu(CH_3_COO)_2_·H_2_O, zinc acetate dehydrate, Zn(CH_3_COO)_2_·2H_2_O (ACS reagent, ≥98%, Sigma-Aldrich), resorcinol (99.9%, Chimlaborreativ, Brovary, Ukraine) and a 37% aqueous solution of formaldehyde ((stabilized with about 10% methanol) for synthesis, Sigma-Aldrich) were used in the synthesis of composites. Double-distilled water was used as the solvent. In the adsorption experiment, 4-nitrophenol and 4-chlorophenol, delivered by Merck (Darmstadt, Germany), were used as adsorbates.

### 2.2. Preparation of Nanocomposites

A typical preparation of M_x_O_y_/SiO_2_ oxide nanocomposites (where M is Cu, Mg, Mn, Ni and Zn) consists of three steps. In the first step, the homogeneous dispersion of silicon dioxide was prepared in the aqueous solution of the corresponding metal acetate, with an estimated ratio of the components under stirring at room temperature. The content of the metals was 3.0 mmol/g SiO_2_. In the second stage, the dispersions were dried at a layer thickness of 4–7 mm at 130 °C for 5 h, then ground in a mortar and sieved through a sieve with a mesh size of 0.5 mm. In the third and final stage, all the powders obtained were calcined in air at 600 °C for 2 h. The reference sample of fumed silica was treated in the same three steps: homogenization of the aqueous dispersion, drying, grinding, sieving and calcination at the same temperature. 

The modification of resorcinol–formaldehyde (RF) polymers by oxide nanocomposites was carried out by an in situ method by mixing resorcinol, formaldehyde and M_x_O_y_/SiO_2_ nanocomposites or pristine SiO_2_ at a weight ratio of 1:2:1 with stirring at room temperature. The unfilled resorcinol–formaldehyde control sample (RFR) was prepared by stirring resorcinol with formaldehyde at the same 1:2 weight ratio. All mixtures were hermetically sealed, placed in a thermostatic oven and treated at 50 °C for 4 days for complete curing and maturation of the RF resin. After gelling, a brown, solid polymer composite was obtained, which was dried at the same temperature for 18 h. All polymer composites were crushed and sieved to obtain a fraction of 0.2 to 0.5 mm. The polymer composites were labeled as RF/SiO_2_, RF/Cu/SiO_2_, RF/Mg/SiO_2_, RF/Mn/SiO_2_, RF/Ni/SiO_2_, RF/Zn/SiO_2_ and RF/Zn/SiO_2_.

The carbonization of the samples was carried out in a tubular furnace under a nitrogen atmosphere (with a flow rate of 100 mL/min) by heating from room temperature to 800 °C at a heating rate of 5 °C/min and holding at the maximum temperature for 2 h. As-synthesized composites were designated as C/SiO_2_, C/Cu/SiO_2_, C/Mg/SiO_2_, C/Mn/SiO_2_, C/Ni/SiO_2_ and C/Zn/SiO_2_.

### 2.3. Characterization Methods

X-ray diffraction (XRD) patterns were recorded at 2θ = 10–70° using a DRON-4-07 (Burevestnik, St.-Petersburg, Russia) with Cu Kα radiation in the geometry of Bragg–Brentano. The average sizes of crystallites were estimated using the Sherrer equation [31].

The Raman spectra were recorded using an inVia Reflex Microscope DMLM Leica Research Grade, Reflex, Renishaw, UK (excitation at 514 nm).

The surface morphology of the samples was studied by field emission scanning electron microscopy (SEM, QuantaTM 3D FEG (FEI, Hillsboro, OR, USA) operating at a voltage of 30.0 kV).

To investigate the textural characteristics, low-temperature (77.4 K) nitrogen adsorption–desorption isotherms were recorded using a Micromeritics ASAP 2405N adsorption analyzer. The specific surface area (*S_BET_*) was calculated according to the standard BET method [32]. The total pore volume *V_p_* was evaluated from the nitrogen adsorption at p/p_0_ = 0.98–0.99 (p and p_0_ denote the equilibrium and saturation pressure of nitrogen at 77.4 K, respectively). The nitrogen desorption data were used to compute the pore size distributions (PSDs, differential *f_V_*(*R*)~d*V_p_*/d*R* and *f*_S_(*R*)~d*S*/d*R*) using a model with slit-shaped cylindrical pores and voids between nanoparticles (SCV) [33]. The differential PSDs with respect to pore volume *f*_V_(*R*)~d*V_p_*/d*R*, ∫*f*_V_(*R*)d*R*~*V_p_* were recalculated as incremental PSDs (IPSD, ∑ Φ_v_,_I_ (*R*) = *V_p_*) = (*R*). The *f*_V_(*R*) and *f*_S_(*R*) functions were also used to calculate contributions of micropores (*V_micro_* and *S_micro_* at *R* ≤ 1 nm), mesopores (*V_meso_* and *S_meso_* at 1 nm < *R* < 25 nm) and macropores (*V_macro_* and *S_macro_* at *R* > 25 nm) to the total pore volume and specific surface area.

Thermal analysis, including thermogravimetry and differential thermal analysis (TG-DTG) (Derivatograph C, MOM, Budapest, Hungary), was used to obtain data on the decomposition processes, thermal stability and temperature of phase transformations of the prepared materials via heating the samples (20–22 mg) in a static air atmosphere from 20 to 1200 °C at a heating rate of 10 °C/min.

The thermal decomposition of samples was determined using a STA 449 Jupiter F1 (Netzsch, Waldkraiburg, Germany) coupled online with a FTIR spectrometer (Bruker, Berlin, Germany). The samples (~12 mg) were heated at a rate of 10 °C/min in the range of 30–1000 °C in an atmosphere of nitrogen (50 mL/min) in an alumina crucible and sensor thermocouple type S TG–DSC. An empty Al_2_O_3_ crucible was used as a reference. The data were recorded and processed using NETZSCH Proteus^®^ software, version 6.1.

### 2.4. Adsorption from Solutions

The composites used in the adsorption experiments were dried for 3 h at 80 °C to remove moisture. Equilibrium adsorption isotherms of p-chlorophenol (ChP) and p-nitrophenol (NP) were determined using the method previously described in the paper [34]. For this purpose, 30 mg of adsorbent was weighed and placed in a 50 mL conical flask, and then 29.9 mL of deionized water and 0.1 mL of the appropriate adsorbate solution were added (*C*_0_ = 7.78 mmol/L (ChP) and *C*_0_ = 7.19 mmol/L (NP)). The mixtures in flasks, tightly closed with corks, were shaken for 48 h in an Innova 42 incubator at a speed of 110 RPM at a temperature of 25 °C. Then, 3 mL of the clear supernatant solution was taken from each sample to measure the absorption spectra using a Cary 100 (Varian) UV-Vis spectrophotometer to determine the equilibrium concentration of phenols. After the measurement, the appropriate amounts of water and the initial solution were added to each flask so that the total volume of the solutions was again 30 mL. The prepared solutions were shaken again under the same conditions as described earlier. The cycles were repeated several times until the adsorption isotherm was completed. The concentration of adsorbates was determined based on absorbance values at λ = 225 nm and λ = 227 nm for p-chlorophenol and p-nitrophenol, respectively. The equilibrium concentration was calculated based on the following equation:

(1) $$a_{eq}=\frac{(C_{0}-C_{eq})\cdot V}{m}$$

where *C*_0_ is the initial adsorbate concentration (mmol/L), *C_eq_* is the adsorbate concentration at adsorption equilibrium (mmol/L), *a_eq_* is the solute adsorbed value per gram of adsorbent (mmol/g), *V* is the volume of the solution (L) and *m* is the adsorbent weight (g).

## 3. Results and Discussion

### 3.1. Thermal Decomposition of Resorcinol–Formaldehyde Polymer in the Presence of Oxide Composites in N_2_ Atmosphere

The thermogravimetric method made it possible to evaluate the thermal stability of composites and provide information on the effect of fillers on the process of polymer decomposition in the nitrogen atmosphere or in air over a wide temperature range. Thermal analyses including thermogravimetry (TG), differential thermogravimetry (DTG) and differential scanning calorimetry (DSC) were performed to understand the thermal decomposition (pyrolysis) and mass loss of the composites in the presence of different silica oxide fillers, as well as to assess the effect of metals on polymer degradation.

**Figure 1:**
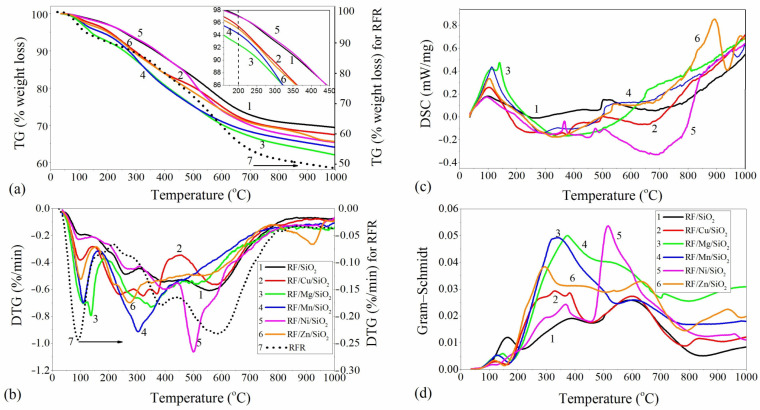
TG-DTG-DSC curves (**a**–**c**) for the polymeric nanocomposites with Gram–Schmidt profile (**d**) (in N_2_ atmosphere).

Concerning the initial RFR polymer, degradation of the crosslinked resorcinol–formaldehyde polymer in the inert atmosphere proceeded in two main stages (Figure 1a,b, dot line): (i) in the temperature range up to 214 °C, where physically absorbed water was removed (mass loss of 10% with *T_max_* = 92 °C); (ii) at 214–860 °C, where polymer decomposition took place. The second part can be divided into low-temperature (214–450 °C) and high-temperature (450–860 °C) stages with breakage of C-O and C-H bonds, respectively, where the pyrolysis of the polymer took place and volatile gasses and carbonaceous char formed [35]. The differential thermogravimetric (DTG) curve indicates where maximum rates of weight loss occurred at each of the temperatures. According to the DTG curve, the maximum weight loss rate occurred at *T_max_* = 89 and 580 °C. The total weight loss is about 51% during carbonization in N_2_ (Table 1).

TG-DTG-DSC curves for polymeric composites modified with oxide fillers, heated from room temperature to 1000 °C under a nitrogen atmosphere, are shown in Figure 1. TG and DTG curves can be divided into several sections vs. temperature typical for the degradation of initial and modified resins [36,37]. The first stage is in the temperature range of 40–220 °C and represents a removal of surface adsorbed volatiles, in particular, physically absorbed water in the modified composites. The Δm values of the first stage varies in the range of 2.8–10.3%. The difference in the values of mass losses can be explained by the increased ability of modified pyrogenic silica Me_x_O_y_/SiO_2_ to retain adsorbed water in surface structures and texture micropores, the contribution of which changes upon the type of metal oxide incorporated into the silica [38]. Moreover, it was shown by Charmas B. et al. that microporous materials have a higher temperature of water evaporation [39]. A double peak in the RF/Mg/SiO_2_ sample and the corresponding mass loss can be attributed to the release of the bonded water due to the increased hydrophilicity of the MgO in the nanocomposite [40].

The second stage of polymer carbonization is in the range of 200–960 °C and can be divided into a low-temperature stage (140–450 °C) and a high-temperature stage (450–860 °C), as in the case of the original RF polymer. At the same time, the temperature of the transformation of the polymer increases in the line Ni < Cu < Zn < Mn < Mg (Figure 1a, Table 1). It should be noted that all samples in the second stage show a non-uniform DTG curve. There are two or more additional peaks that occur near the maximum. Considering the asymmetric shape of the peaks in the second stage, it can be suggested that it is a multistage overlapping process involving different decomposition reactions in the carbonization process. Figure 1 and Table 1 show that the main rate of weight loss for Cu-, Mg-, Mn- and Zn-containing composites took place in the low-temperature stages, which can be assigned to the thermal transformation and decomposition of RF during the formation of a carbon skeleton, whereas, in Ni- and SiO_2_-containing samples, the main mass losses are observed in the second high-temperature stage. The total mass loss of metal-containing composites was higher than that for RF/SiO_2_. The structure of the carbon matrix is ordered in the range of 800–1000 °C.

**Figure 2:**
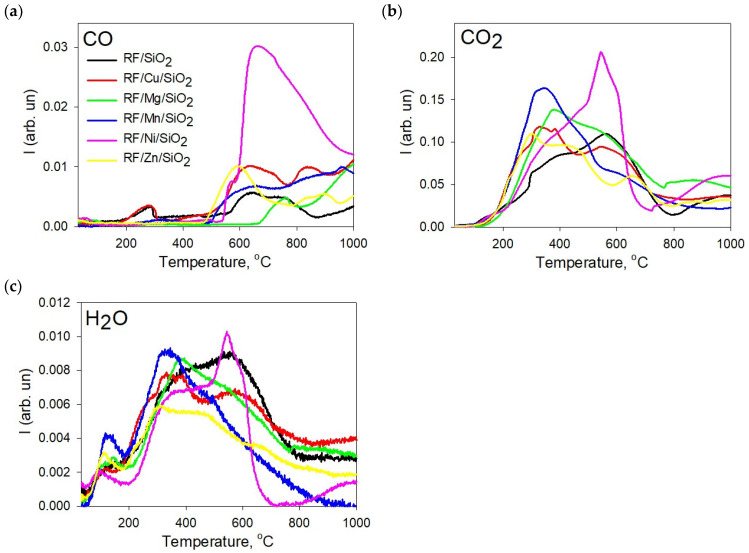
TPD spectra of the main gaseous products of pyrolysis of resorcinol–formaldehyde resin modified with metal oxide/silica nanocomposites: CO (**a**), CO_2_ (**b**), H_2_O (**c**).

The slight increase in mass (Figure 1a, TG curve) in the Ni-containing sample in the range from 200 to 500 °C is related to a decrease in the oxidation state of the metal under the action of polymer pyrolysis products and the formation of metallic Ni, which is confirmed by XRD data (discussed below, Figure 2, Table 2). The slow decrease in mass in the RF/Zn/SiO_2_ sample at temperatures above 860 °C and up to 1000 °C at T_max_ = 925 °C (DTG curve) can be attributed to structural transformations in the silicate structures formed (Figure 2). The relatively high residue of the composites indicates that thermally stable decomposition products are formed after pyrolysis (Table 1, Δm_total_).

The DSC analysis agrees with the TGA results, which indicate that exothermic reactions took place above 200 °C. This could be due to the formation of C-C bonds at the expense of C-O bond breakage. The DTG peaks above 450 °C are consistent with the inflection point in the DSC curve (Figure 1c).

Fourier transform infrared spectroscopy (FT-IR) was used to understand the phenomena of weight loss and gas emitting during pyrolysis. The changes in the exhaust gasses during pyrolysis were plotted using a Gram–Schmidt diagram. The dependence of the FTIR absorption intensity of the gasses on the temperature was plotted in Figure 1d. The Gram–Schmidt profile should be similar to the DTG curve and reflect the correlation between the mass loss and the gas evolution detected by the FT-IR spectrometer. However, in our case, a shift of 20 °C was observed in the FT-IR spectra compared to the main picks of the DTG curve. This shift reflects the transport of evolved gasses from the sample into the gas cell of the FT-IR spectrometer (an inherent delay until the FT-IR spectrum is acquired) [41]. The FT-IR spectra show bands assigned to the rotational vibrational frequencies of water in the vapor phase (1300–2000, 3400–4000 cm^−1^) up to 100 °C. With a further increase in temperature, bands characteristic of CO_2_ appeared in the spectra (600–750 cm^−1^) and two sharp peaks with high intensity at 2358 and 2321 cm^−1^ are visible (Figure S1). No other detectable organic species (phenol, formaldehyde, benzene) were released from the sample.

The nature of the change in the intensity of these peaks until the end of the heat treatment is different for each sample, indicating that the metals have a significant influence on the process of polymer pyrolysis. In Figure 2, the TPD spectra of the main gaseous products of the pyrolysis of resorcinol–formaldehyde resin modified with metal oxide/silica nanocomposites are presented. It should be noted that H_2_O is released over the entire temperature range. The spectra show CO as an additional product released by the thermal decomposition of the phenol groups. It is noticeable that the emission of CO gas at 150 °C is higher from the RF/SiO_2_ sample (Figure S1) than for the modified polymer composites, while the temperature at which it occurs is shifted to the high-temperature range and increases by 100 °C in the series Ni < Cu < Mg < Zn < Mg. Thus, the main volatile products during the pyrolysis of the resorcinol–formaldehyde polymer are CO, CO_2_ and H_2_O. According to the bond strength data, these decomposition reactions may involve the breaking of C-O bonds at the lower temperature and the breaking of C-H bonds at the higher temperature [42]. The main weight loss at this stage begins with the breaking of the methylene bonds of the main chains with the simultaneous release of carbon dioxide and carbon monoxide in the form of gaseous products. They can be formed by the complete decomposition of the benzene ring, which can take place at up to 1200 °C. This cracking process leads to the breakdown of small fragments and the formation of free radicals. These free radicals are then either recombined or abstracted from the three-dimensional hydrogen or oxygen residue, which gradually leads to an increasing carbonization of the residue. It is confirmed that the degradation of the three-dimensional network begins by the breakdown of methylene bridges, followed by breaks in the phenolic functions [43].

The process of the thermal destruction of a cross-linked solid resorcinol–formaldehyde polymer with constant temperature increase is accompanied by continuous changes in the structural fragments caused by chemical transformations in the polymer chains. The developing processes of the cleavage of methylene groups as well as condensation and removal of water reduce the mobility of the polymer chains and contribute to the formation of diverse and unequal structures that determine a wide temperature range for the release of H_2_O, CO_2_ and CO.

The production of CO and CO_2_ in Ni-containing samples was intense and CO_2_ was shifted to higher temperatures. The differences in the yield of gaseous CO and CO_2_ products were due to the nature of the polymer (structure, cross-linking and curing) rather than with its composition, which is consistent with the literature data [44].

The shape of the curve allows us to determine several stages of H_2_O release (Figure 2) that are characteristic of all samples. The first stage is at 200 °C; the second stage is characterized by an increase in water release in the 200–500 °C range; in the third stage, the intensive formation and release of water continues up to a temperature of 700 °C. A significant difference is the intensity of H_2_O evolution in the Mg_x_O_y_/SiO_2_ sample in the first two stages at temperatures up to 200 °C and 400 C. This fact can be explained by the increased ability of modified pyrogenic silica Mg_x_O_y_/SiO_2_ to retain adsorbed water both in surface structures and textured microparticles.

### 3.2. Porosity of Composites

**Figure 3:**
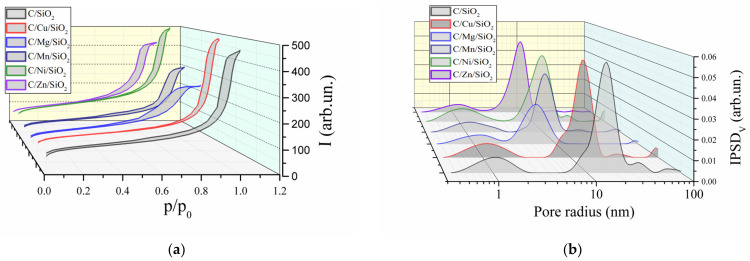
(**a**) Nitrogen adsorption/desorption isotherms (lower rising/upper falling curves, respectively) and (**b**) pore size distribution of carbon nanocomposites.

According to the IUPAC classification, the isotherm of C/M/SiO_2_ nanocomposites is type IV (Figure 3) with an H3 type hysteresis loop, indicating the presence of slit-shaped mesopores in the matrix with a non-uniform size. The shapes of pore size distributions also presented in Figure 3 show the formation of a uniform system of mesopores. The determined values of parameters characterizing the pore structure (BET surface area, pore areas and pore volumes) of the carbon composites are presented in Table 2. As can be seen, the main part of the porosity is formed by mesopores, and the share of micro- and macropores is small. Nitrogen adsorption/desorption isotherms and PSDs of initial M_x_O_y_/SiO_2_ composites are described elsewhere [25].

As can be seen according to the given data, the carbon phase is formed in the textural pores of the oxide matrix, significantly reducing the volume of the mesopores, as well as on the surface of the dispersed oxide aggregates, forming macropores. The carbon in the mesopores can form aggregates as was presented in the paper [25], forming additional slit-shaped pores. Its content in the samples was 36–48% by weight. Samples of carbon oxide nanocomposites in the presence of nickel and copper have the highest porosity and largest specific surface area.

### 3.3. Structural and Morphological Analysis

**Figure 4:**
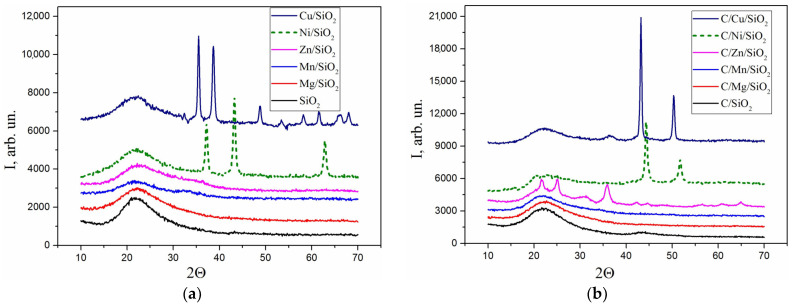
X-ray diffraction patterns of MxOy/SiO_2_ (**a**) and carbonized C/M/SiO_2_ (**b**) nanocomposites.

The thermo-oxidative destruction of the adsorbed complex of metal acetates leads to the formation of amorphous or crystalline oxide nanoparticles in the interstices between the primary particles of the aggregates and the associations of fumed silica. The structure and phase composition of the oxide fillers and the carbonized C/M/SiO_2_ nanocomposites are summarized in Table 3 and shown in Figure 4. According to the obtained XRD data, the formation of nanocrystalline oxide phases of CuO (JCPDS No. 48-1548) and NiO (JCPDS No. 47-1049) was observed only in Ni- and Cu-containing nanocomposites after heating at 600 °C, while composites with Mg, Mn and Zn were amorphous when using the same concentration of metal salts (3 mmol/g SiO_2_). The concentration of adsorbates of 3 mmol/g SiO_2_ was used in the present work, since the formation of adsorption complexes with Si-OH groups results in an almost complete, uniform coverage of the surface of the silica matrix with adsorbed acetates. The presence of the silica matrix thus limits the growth of the metal oxide nanoparticles after calcination and prevents their agglomeration to obtain nanoparticles with a more dispersed and uniform distribution in the carbon mass.

X-ray diffraction patterns of all carbonized composites indicate the presence of a broad halo in the region of 2θ = 22° characteristic for amorphous silica. Furthermore, it practically coincides in an angular position with the first halo of the disordered structure of the carbon material (Figure 4). The second one is clearly observed at 2θ = 40–50° in the C/SiO_2_ samples. For the zinc-containing composite, the formation of crystalline silicate β-Zn_2_SiO_4_ (JCPDS 14-653) was observed under pyrolysis conditions. The diffraction pattern of C/Ni/SiO_2_ shows the presence of metallic nickel (JCPDS No. 4-850) in the char, while, in the C/Cu/SiO_2_ sample, a mixture of metallic copper (JCPDS No. 4-836) and copper(I) oxide JCPDS No. 05-0667) was identified. Peaks of crystalline carbon, namely graphite, were not recorded.

**Figure 5:**
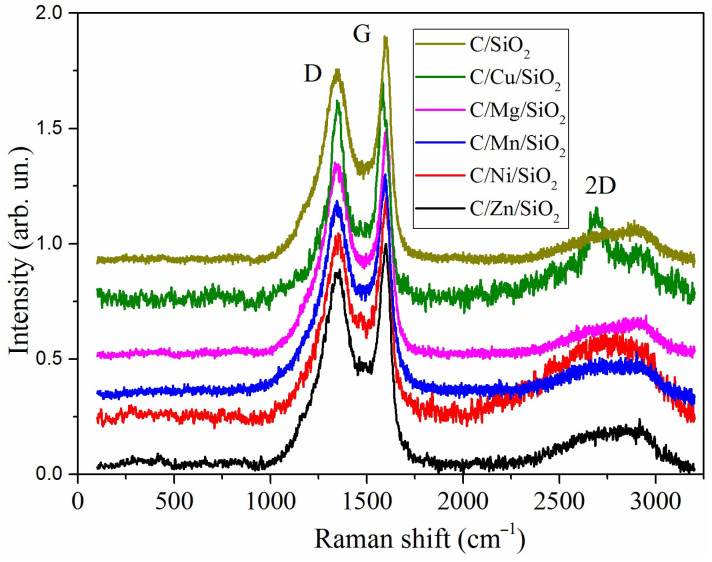
Raman spectra of the carbonized nanocomposites.

In order to determine the structural characterization of the carbon matrix in the nanocomposites, the Raman spectra of the samples were collected (Figure 5). The position of the main bands D (1350 cm^−1^) and G (1582 cm^−1^) and their full width at half maximum (FWHM) were determined (Table 3) [45]. It is also known that, in graphitic sp^2^ materials, there is an additional band at ~2700 cm^−1^, known as the 2D band (or G’ band), which is well registered in Cu- and Ni-containing samples. The G band is a tangential shear mode of carbon atoms that corresponds to the in-plane bond stretching mode E_2g_ of the sp^2^ C–C bond and is characteristic of ordered graphite-like crystallite structures. The origin of the D band is multifaceted. It can arise from the C-C stretching vibration of sp3 carbon atoms, or it may stem from symmetry deviations due to the finite dimensions of crystallites [46]. Additionally, the C-C stretching vibration of carbon atoms, which exhibits a mixed sp^2^-sp^3^ character, can also contribute to the complexity of the D band [47]. The positions of the G peak and the I_D_/I_G_ ratio change depending on the content of sp3 carbon atoms during the transition from ordered graphite to nanocrystalline and amorphous carbon [45]. The shift in the position of the G band to higher frequencies occurred in all samples except for the nanocomposite containing Ni (1582 cm^−1^). It can be attributed to the structural disordering of carbon during the carbonization of the polymeric materials in the presence of different metals or a decrease in the average size of graphite-like structures. Taking into account the changes in the I_D_/I_G_ values, it can be concluded that the ordering of the carbon phase is increased in the line Cu < Mn < Mg < SiO_2_ < Zn < Ni. This is also confirmed by the increase in the FWHM values of the D band and its shift to a lower wavenumber (Table 1, Mg- and Mn-containing samples). The presence of a 2D band in the Cu-containing sample with high disorder of the carbon phase (I_D_/I_G_ ratio) can be explained by the simultaneous presence of both the oxide phase and the reduced metallic copper. The same results were previously obtained with other polymer systems in which the formation of a nanographite shell was observed in the presence of reduced copper [48]. It can therefore be pointed out that the formation of nanocrystalline graphite and amorphous carbon phases can be observed in the nanocomposites.

**Figure 6:**
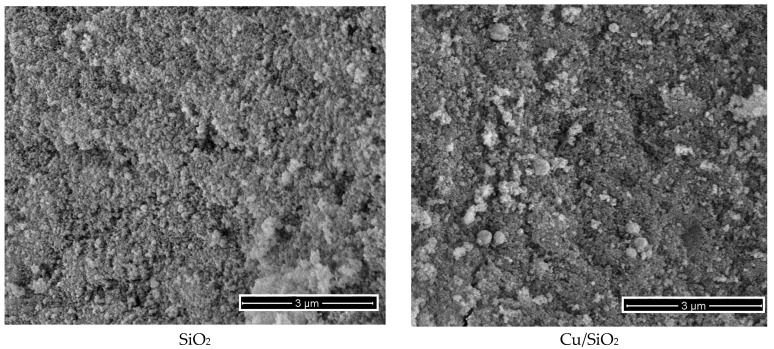

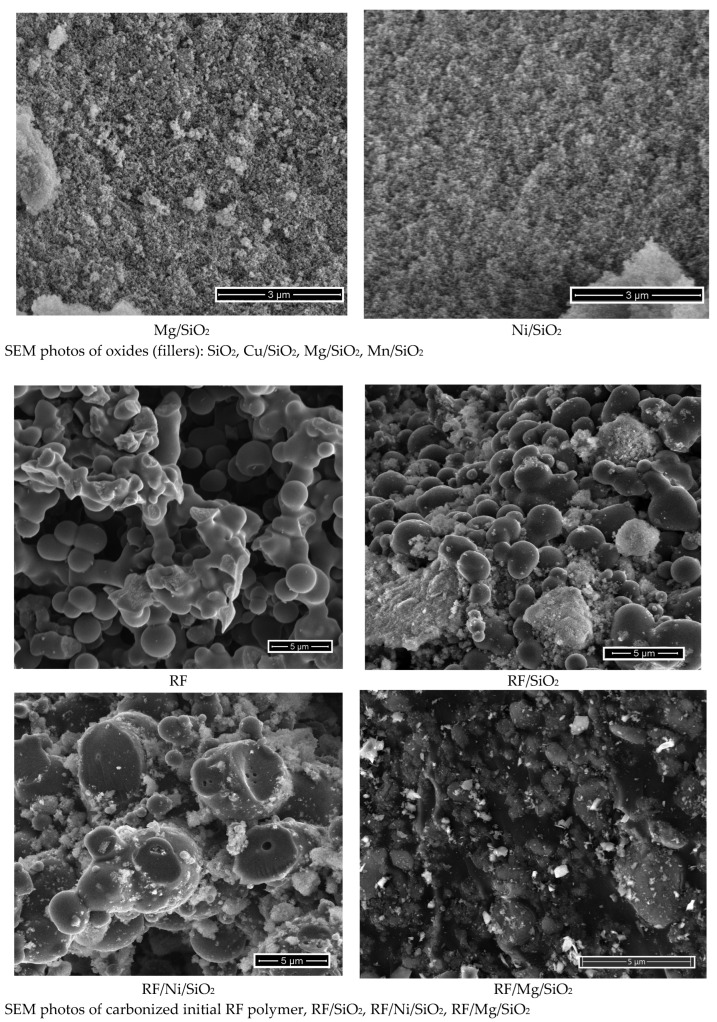
SEM images for the selected materials.

The surface morphology of the obtained materials was studied by scanning electron microscopy and the obtained SEM images are presented in Figure 6. The SEM images of the pristine carbon composite show microspheres with a diameter of about 2–4 μm, which are connected to each other in a chain-like formation and are characterized by a smooth texture and uniform dimensions. In contrast, the SEM images of the altered carbon materials show microspheres that vary in size and have an irregular spherical shape. They are more densely agglomerated and show traces of an amorphous silica layer on their outer surface. It is important to note that the size range of the spheres shows greater variability and has more damaged agglomerates with cracks and pores. In the RF/Mg/SiO_2_ sample, the formation of agglomerates with a denser structure is observed, which, in turn, explains the decrease in the specific surface area of the sample.

### 3.4. Degradation of Composites in Oxidizing Atmosphere

**Figure 7:**
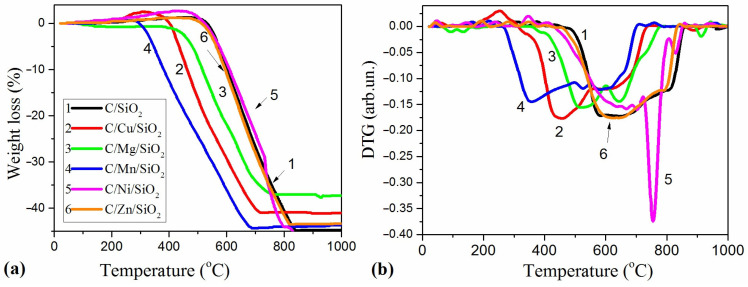
(**a**) TG and (**b**) DTG curves of carbonized C/M/SiO_2_ composites.

A thermal analysis was also performed for the investigated materials in a synthetic air atmosphere. TG and DTG curves of carbonized C/M/SiO_2_ samples are shown in Figure 7. The thermograms of all nanocomposites show an increase in the mass of the samples at temperatures range of 230–650 °C (Figure 7a). This is explained by the oxidation of the carbon particles with the formation of structural oxygen groups on the surface and the beginning of oxidation of the surface of the smallest metal particles at a temperature higher than 230 °C. With a further increase in temperature, two essential processes take place: the oxidation of the metal with the formation of metal oxides, which is accompanied by an increase in the mass of the sample, and the oxidation of the carbon, which leads to the formation of volatile products and a decrease in the mass of the sample. The balance between these two processes, whose occurrence is influenced by the nature of the metals and the structure of the composites, is reflected in the shape of the TG curves (Figure 7). Apart from that, silica remains in residue.

In carbonized Cu- and Ni-containing nanocomposites, a significant increase in the mass of the samples is observed during oxidation in air at temperatures of 250–450 °C, which is related to the oxidation of dispersed Ni and Cu metal via the addition of oxygen and formation of oxide phases (Figure 7). The formation of metallic Cu and Ni was confirmed by XRD analysis (Figure 4, Table 3).

It is known that metals are catalysts for the formation of highly ordered carbon structures. In particular, Ni and Co were previously shown to promote the formation of the graphite phase in the carbonization of organic substances by enhancing the formation of a more ordered nanographite phase [48]. In the present series, Mn and Cu were shown to most actively influence the thermo-oxidative destruction of carbon (Figure 7b). The temperatures of oxidative destruction decreased by 150–230 °C compared to the control sample C/SiO_2_. According to the maximum rates of weight losses on the DTG curve, it can be concluded that the promotion of metals in the catalytic oxidation of carbon increases in the line Mn < Cu < Mg < SiO_2_ < Zn < Ni. The calculated carbon content in the samples was 36–48 wt.%. In addition, the obtained data on the carbon content in the samples indicate that, in a number of metal-containing samples, nickel and zinc promote the formation of the carbon phase (Table 4).

### 3.5. Adsorption Properties

**Figure 8:**
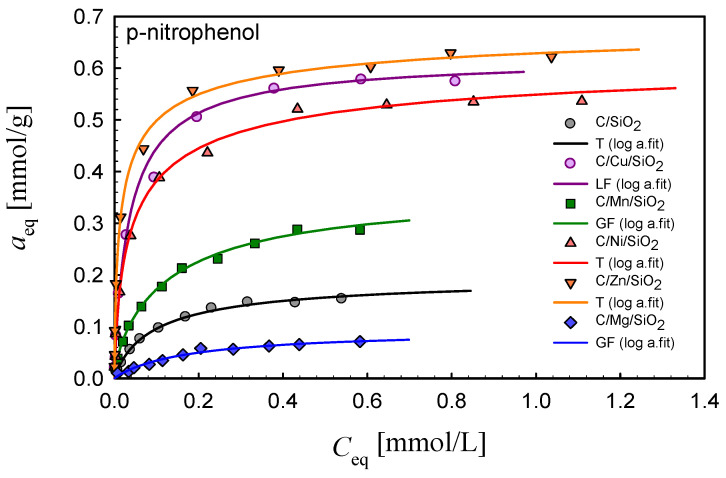
Comparison of the adsorption isotherms for p-nitrophenol on the studied composites. Lines represent the fitted Marczewski–Jaroniec (M-J) isotherm (the so-called generalized Langmuir, GL isotherm).

**Figure 9:**
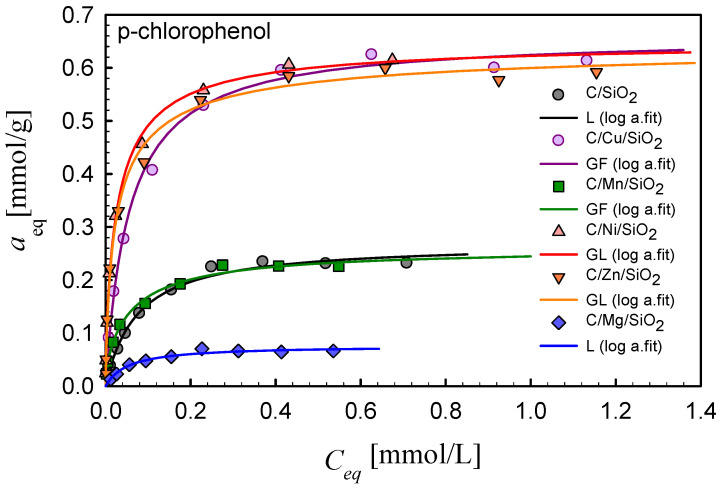
Comparison of the adsorption isotherms for p-chlorophenol on the studied composites. Lines represent the fitted Marczewski–Jaroniec (M-J) isotherm (the so-called generalized Langmuir, GL isotherm).

In order to estimate the adsorption properties of the synthesized composites toward aromatic compounds, the adsorption isotherms of 4-nitrophenol (M_w_ = 139.11 g/mol, pK_a_ = 7.25, c_s_ = 12.1 g/L) and 4-chlorophenol (M_w_ = 128.55 g/mol, pK_a_ = 9.38, c_s_ = 27.1 g/L) were measured. Analyzing the isotherms presented in Figure 8 and Figure 9, one can generally find that the metal nanoparticles promote the adsorption of selected organics in the case of most systems in comparison to the composite C/SiO_2_. The only exception is found for the composite containing magnesium, C/Mg/SiO_2_. For all investigated systems, the maximum adsorption values are differentiated in the range ~0.1–~0.7 mmol/g depending on the metal nanoparticles incorporated into the composite structure. Such differentiation in adsorption uptakes for the synthesized composites indicates the possibility of designing materials of divergent adsorption selectivity adopted for specific applications.

The obtained adsorption equilibrium data were analyzed by using the Marczewski–Jaroniec (M-J) isotherm (often called the generalized Langmuir, GL, isotherm) [49,50]. This theoretical isotherm describes localized physical adsorption from solutions onto an energetically heterogeneous solid surface:

(2) $$\theta =\frac{a_{eq}}{a_{m}}={\left[\frac{{\left(K\cdot \;C_{eq}\right)}^{n}}{1+{\left(K{\;\cdot C}_{eq}\right)}^{n}}\right]}^{\frac{m}{n}}$$

where *θ*—the relative adsorption; *a_m_*—the adsorption capacity [mmol·g^−1^]; *m*, *n*—the heterogeneity parameters characterizing the profile of the adsorption energy distribution function (0 < *m*, *n* ≤ 1); *K*—the adsorption equilibrium constant associated with the characteristic energy of the energy distribution function. The GL isotherm can be reduced to simpler isotherm equations: the generalized Freundlich isotherm (GF; *n* = 1), Langmuir–Freundlich (LF; *m* = *n*), Tóth (T; *m* = 1) and Langmuir (L; *m* = *n* = 1).

In Table 5 and Table 6, the parameters of the GL isotherm are compared for all adsorption systems. As can be seen, the fitting quality is very good, which is confirmed by the values of SD and R^2^. The values of heterogeneity parameters, m, n, indicate moderate- or low-energetic heterogeneity effects. It should also be indicated that the values of the adsorption capacity are in good agreement with the maximum adsorption values.

Taking into account the efficiency of the adsorption process, the composites can be ranked as follows: C/Zn/SiO_2_ (*a_m_* = 0.67) > C/Cu/SiO_2_ (*a_m_* = 0.62) > C/Ni/SiO_2_ (*a_m_* = 0.58) > C/Mn/SiO_2_ (*a_m_* = 0.36) > C/SiO_2_ (*a_m_* = 0.19 > C/Mg/SiO_2_ (*a_m_* = 0.09) (p-nitrophenol) and C/Cu/SiO_2_ (*a_m_* = 0.66) ~ C/Ni/SiO_2_ (*a_m_* = 0.65) ~ C/Zn/SiO_2_ (*a_m_* = 0.62) > C/Mn/SiO_2_ (*a_m_* = 0.25) ~ C/SiO_2_ (*a_m_* = 0.27) > C/Mg/SiO_2_ (*a_m_* = 0.07) (p-chlorophenol). In order to find some correlations between the adsorption effectiveness and adsorbent properties, two factors should be taken into account: textural and surface characteristics of the synthesized composites. Let us first regard the pore structure properties: the values of the specific surface area, *S_BET_*, and total pore and mesopore volumes, *V_p_* and *V_meso_*. Taking into account these parameters, the composites may be ranked as follows: C/Ni/SiO_2_ (*S_BET_* = 443, *V_p_* = 0.795, *V_meso_* = 0.666) > C/Cu/SiO_2_ (*S_BET_* = 377, *V_p_* = 0.786, *V_meso_* = 0.649) > C/Zn/SiO_2_ (*S_BET_* = 333, *V_p_* = 0.638, *V_meso_* = 0.597) > C/Mn/SiO_2_ (*S_BET_* = 323, *V_p_* = 0.532, *V_meso_* = 0.455) > C/Mg/SiO_2_ (*S_BET_* = 311, *V_p_* = 0.443, *V_meso_* = 0.385). Comparing the changes in textural properties with the changes in adsorption effectiveness, one can find a clear correlation between the porosity development and adsorption uptake. The lowest adsorption on the composite C/Mg/SiO_2_ (*S_BET_* = 368, V*_p_* = 0.741, *V_meso_* = 0.633) needs additional explanation because an about 7–8 times lower adsorption capacity between this material and C/Zn/SiO_2_ cannot be simply related to a poorly developed internal pore space (*S_BET_* lower by only 20 m^2^/g, pore volume lower by 30%). In this case, we should regard the influence of surface active sites originating from incorporated metal nanoparticles. Analyzing the TG and DTG curves (Figure 1 and Figure 5), we found a differentiation in the amount of physically absorbed water in the composites containing different metal oxides. This is explained by the increased and divergent ability of modified pyrogenic silica Me_x_O_y_/SiO_2_ to retain adsorbed water in surface structures and micropores depending on the type of metal oxide incorporated into the silica [38]. This effect is the strongest for Mg-containing materials as a result of the increased hydrophilicity of MgO in the nanocomposite [40]. Taking into account the larger amounts of adsorbed water with the less developed porous structure of C/Mg/SiO_2_, a significant reduction in the pore volume/area available for adsorbates should be expected, which results in a decrease in adsorption capacity.

Generally, the creation of metallic active sites should be indicated as a very important factor determining the composite adsorption effectiveness. Relatively low adsorption on the composite without metal nanoparticles, C/SiO_2_, with well-developed porosity confirms this observation. In this case, the hydrophobic interactions between the carbon layers and aromatic solutes are the dominating adsorption mechanism. Thus, a much lower adsorption uptake for this system indicates another adsorption mechanism for metal-containing materials: interactions between solutes and metallic active sites as the additional adsorption centers. Such centers can change the acid/base character of the adsorbent surface; moreover, their high ability to react with water molecules, resulting in the creation of surface groups, -M–OH, should also be taken into account. Depending on the solution pH and ionic/neutral form of the adsorbate, the mechanism of electrostatic interactions should also be regarded.

## 4. Conclusions

The proposed method has the advantage that the presence of a solid matrix can limit the growth of metal nanoparticles and thus the size of the metal nanoparticles can be controlled. This prevents the agglomeration of metal nanoparticles, resulting in nanoparticles with a more dispersed and uniform distribution in the mesoporous carbon. In addition, the synthesized mesoporous carbon has a large specific surface area and a high degree of order.

During the carbonization process, the carbon species originating from the polymer are catalytically decomposed, leading to the formation of different carbon structures. The specific catalytic activities of the metals used in the different composites lead to different formation mechanisms. These differences have a significant effect on the pyrolysis process and consequently influence the composition of the carbon, the metal particles and the resulting composite structures.

It has been shown that the presence of nickel (Ni) and zinc (Zn) plays a positive role in the formation of the graphite phase during the carbonization process of organic substances. In particular, their influence leads to the formation of a more ordered nanographite phase, which contributes to the overall structural properties of the material.

The samples of carbon oxide nanocomposites in the presence of nickel and copper exhibit the largest pore volume and specific surface area. Indeed, the data show an interesting phenomenon: during the carbonization process, the carbon phase forms in the structural pores of the oxide matrix. This process leads to a considerable reduction in the volume of the mesopores. Interestingly, macropores also form on the surface of the dispersed oxide aggregates. These structural changes have an impact on the properties and applications of the material.

For most investigated materials with incorporated metal nanoparticles, one can find a strong increase in adsorption effectiveness toward p-chlorophenol and p-nitrophenol in comparison to the carbon/silica composite. The only exception is found for the composite containing magnesium, C/Mg/SiO_2_. Generally, for all investigated systems, the maximum adsorption values are differentiated in the range ~0.1–~0.7 mmol/g depending on the metal nanoparticles incorporated into the composite structure, and they can be ranked as follows: C/Zn/SiO_2_ > C/Cu/SiO_2_ > C/Ni/SiO_2_ > C/Mn/SiO_2_ > C/SiO_2_ > C/Mg/SiO_2_ (p-nitrophenol) and C/Zn/SiO_2_ ~ C/Cu/SiO_2_ ~ C/Ni/SiO_2_ > C/Mn/SiO_2_ ~ C/SiO_2_ > C/Mg/SiO_2_ (p-chlorophenol). The divergent selectivity of synthesized composites was related to textural parameters and surface properties connected with the creation of new active sites originating from metal nanoparticles. Further research should be focused on designing materials of divergent adsorption selectivity adopted for specific applications.

## Figures and Tables

**Table 1:** Thermogravimetry data for polymer degradation.

Sample	Temperature Range, I Stage, °C	T_maxI_ °C	Δm_I_, %	Low- Temperature Range, °C	T_max_ °C	Δm, %	High- Temperature Range, °C	Δm_I_, %	T_maxII_ °C	Δm_total_, %
RF	35–217	89	10.3	215–445	385	13.5	446–800	38	580	51
RF/SiO_2_	35–210	102.7	4.1	141–411	258.5 395.9	10.8	412–800	17.3	556	30
RF/Cu/SiO_2_	35–150	98.7	2.8	144–455	237.6 321.1 371.4	16.5	456–800	12.8	582	32.1
RF/Mg/SiO_2_	35–184	137.6	5.3	184–472	351.4	18.1	472–800	11.6	510	38.1
RF/Mn/SiO_2_	35–168	110.3	4.7	168–467	304	17.6	467–800	11.3	-	36.1
RF/Ni/SiO_2_	35–203	93.2	3.2	203–445	267.4 399.2	11.2	445–800	18.8	500.2	34.6
RF/Zn/SiO_2_	35–160	100.8	3.5	156–485	221.3 274.8	17.1	486–800	11.2	520.9 921	34.4

*T_max_*,—temperature at the maximum degradation rate of individual stages, Δ*m*—mass loss in determined temperature range.

**Table 2:** Textural properties of pyrolyzed carbonaceous nanocomposites.

Sample	*S_BET_* (m^2^/g)	*S_micro_* (m^2^/g)	*S_meso_* (m^2^/g)	*S_macro_* (m^2^/g)	*V_p_* (cm^3^/g)	*V_micro_* (cm^3^/g)	*V_meso_* (cm^3^/g)	*V_macro_* (cm^3^/g)
C/SiO_2_	368	181	185	1.2	0.741	0.075	0.633	0.033
C/Cu/SiO_2_	377	202	172	3.2	0.786	0.078	0.649	0.059
C/Mg/SiO_2_	311	119	192	0.2	0.443	0.050	0.385	0.008
C/Mn/SiO_2_	323	153	169	0.7	0.532	0.060	0.455	0.017
C/Ni/SiO_2_	443	215	226	2.1	0.795	0.086	0.666	0.043
C/Zn/SiO_2_	333	118	214	0.7	0.638	0.046	0.597	0.013

**Table 3:** Structure and phase composition of oxide fillers and carbonized C/M/SiO_2_ nanocomposites.

Sample	Filler, MxOy/SiO_2_		Carbonized Nanocomposites, C/M/SiO_2_						
	Phase Composition	Size, nm	Phase Composition	Size, nm	D Band, cm^−1^	G Band, cm^−1^	FWHM		I_D_/I_G_
							D	G	
C/SiO_2_	SiO_2_ *_amorph_*	–	SiO_2_ *_amorph_*	–	1356	1596	142.9	79.1	0.89
C/Cu/SiO_2_	CuO SiO_2_	22 –	Cu, Cu_2_O SiO_2_ *_amorph_*	–	1352	1600	148.8	71.3	1.0
C/Mg/SiO_2_	SiO_2_ *_amorph_*	–	SiO_2_ *_amorph_*	–	1343	1594	157.0	68.0	0.97
C/Mn/SiO_2_	amorph.	–	MnO, SiO_2_ *_amorph_*	–	1345	1598	160.0	69.0	0.98
C/Ni/SiO_2_	NiO SiO_2_ *_amorph_*	17 –	Ni, SiO_2_ *_amorph_*,	–	1350	1582	110.8	77.1	0.75
C/Zn/SiO_2_	amorph	–	SiO_2_ *_amorph_*, Zn silicate β-Zn_2_SiO_4_	–	1351	1598	140.6	63.1	0.78

**Table 4:** Carbon content in the samples.

Sample	C/SiO_2_	C/Ni/SiO_2_	C/Zn/SiO_2_	C/Mn/SiO_2_	C/Cu/SiO_2_	C/Mg/SiO_2_
C content, wt%	45.95	47.66	44.91	44.80	43.39	36.33

**Table 5:** Parameters of the generalized Langmuir equation for the adsorption of p-nitrophenol on the studied composites.

Adsorption System	Isotherm Type	*a_m_*	*m*	*n*	log *K*	*R* ^2^	*SD*(*a*)
C/SiO_2_	T	0.19	1	0.8	1.11	0.999	0.001
C/Cu/SiO_2_	LF	0.62	0.90	0.90	1.44	0.987	0.017
C/Mn/SiO_2_	GF	0.36	0.65	1	0.66	0.997	0.002
C/Ni/SiO_2_	T	0.58	1	0.57	1.91	0.976	0.022
C/Zn/SiO_2_	T	0.67	1	0.52	2.38	0.995	0.019
C/Mg/SiO_2_	GF	0.09	0.93	1	0.71	0.976	0.004

*a_m_*—adsorption capacity [mmol·g^−1^]; *m*, *n*—heterogeneity parameters; log*K*—logarithm of the equilibrium constant related to characteristic adsorption energy; *R*^2^—determination coefficient; *SD*(*a*)—standard deviation [mmol·g^−1^].

**Table 6:** Parameters of the generalized Langmuir equation for the adsorption of p-chlorophenol on the studied composites.

Adsorption System	Isotherm Type	*a_m_*	*m*	*n*	log *K*	*R* ^2^	*SD*(*a*)
C/SiO_2_	L	0.27	1	1	1.13	0.991	0.008
C/Cu/SiO_2_	GF	0.66	0.88	1	1.15	0.994	0.019
C/Mn/SiO_2_	GF	0.25	0.58	1	0.96	0.993	0.008
C/Ni/SiO_2_	GL	0.65	0.63	0.88	1.35	0.998	0.015
C/Zn/SiO_2_	GL	0.62	0.74	0.86	1.46	0.963	0.048
C/Mg/SiO_2_	L	0.07	1	1	1.27	0.969	0.004

*a_m_*—adsorption capacity [mmol·g^−1^]; *m*, *n*—heterogeneity parameters; log*K*—logarithm of the equilibrium constant related to characteristic adsorption energy; *R*^2^—determination coefficient; *SD*(*a*)—standard deviation [mmol·g^−1^].

## Data Availability

The data are available by the corresponding author.

## Supplementary Materials

The following supporting information can be downloaded at: https://www.mdpi.com/article/10.3390/ma17091981/s1, Figure S1: FTIR spectrograms for the combustion of the samples; Table S1: Summarized results of the FTIR analysis for the most important peaks in Figure S1.
