# Supplementary material for: Physicochemical and Adsorption Characterization of Char Derived from Resorcinol–Formaldehyde Resin Modified with Metal Oxide/Silica Nanocomposites
Source: Materials (Basel). 2024 Apr 24;17(9):1981. doi: 10.3390/ma17091981 (PMC11084562; doi:10.3390/ma17091981)
Supplement: Supplementary file 1 [file materials-17-01981-s001.zip › materials-2965250-supplementary.pdf]

materials

MDPI

Supplementary Material

Characterization of char derived from resorcinol-formaldehyde resin modified with metal oxide/silica nanocomposites

Mariia Galaburda[1,2], Dariusz Sternik[1], *Agnieszka* Chrzanowska[1], Olena Oranska[2], Yurii Kovalov[3], Anna Derylo-Marczewska[1]

[1] Faculty of Chemistry, Maria Curie-Skłodowska University, Maria Curie-Sklodowska Sq.3, 20-031 Lublin, Poland; mariia.galaburda@gmail.com (M.G); dariusz.sternik@mail.umcs.pl (D.S.); agnieszka.chrzanowska@mail.umcs.pl (A.C.); anna.derylo-marczewska@mail.umcs.pl (A.D.-M.)
[2] Chuiko Institute of Surface Chemistry, 17 General Naumov Str., Kyiv 03164, Ukraine; mariia.galaburda@gmail.com (M.G.); el.oranska@gmail.com (O.O)
[3] School of Chemistry, University of Bristol Cantock's Close, Bristol, BS8 1TS (UK); ununulium123@gmail.com (Y.K.)
* Correspondence: anna.derylo-marczewska@mail.umcs.pl (A.D.-M.) Tel.: +48-8153-755-49; mariia.galaburda@gmail.com (M.G.)

It was noted, that in the case of degradation under nitrogen for the Ni samples, the peaks corresponding to CO_2 is broad and present two maxima at 350 and 550C.

The stretch vibration bands detected at 1150-1085 cm^{-1} in RF-SiO_2 correspond to the C-O stretching in the aliphatic ether.

The absorbance of –OH stretching at 4000–3500 cm^{-1} is associated with H_2O and the phenolic hydroxyl group.

The stretch vibration bands detected at 3000-2500 cm^{-1} correspond to the C–H group in the aldehyde or ketone compounds.

The absorbance of –OH stretching at 4000–3500 cm^{-1} is associated with H_2O and the phenolic hydroxyl group.

Table S1. Summarized results of the FTIR analysis for the most important peaks in Figure S1.

Functional group	Wave number, cm–1
CO_2	600-750, 2400–2240, 3900-3500
CO	1950-2150

Citation: To be added by editorial staff during production.

Academic Editor: Firstname Lastname

Received: date
Revised: date
Accepted: date
Published: date

Copyright: © 2024 by the authors. Submitted for possible open access publication under the terms and conditions of the Creative Commons Attribution (CC BY) license (https://creativecommons.org/licenses/by/4.0/).

H_2O	1300-2000, 3400-4000
C–H	3000–2830
C=O	1850–1600
C=C	1600–1420

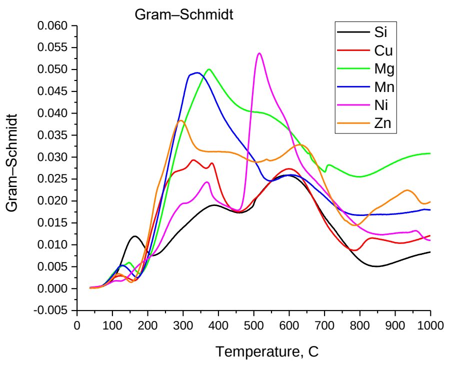

Gram-Schmidt curves corresponding to the TGA-FTIR analysis of composites in N_2.

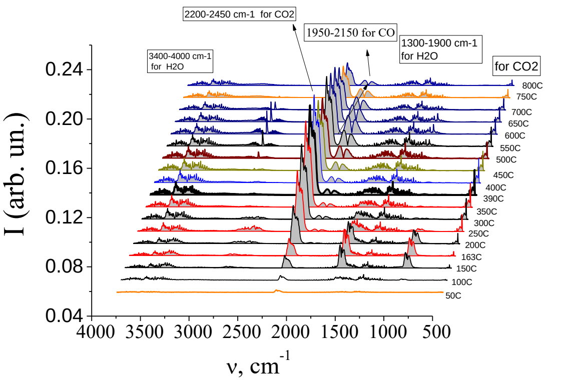

RF-SiO_2

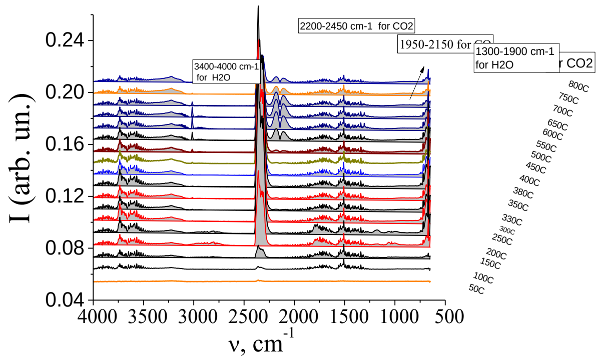

RF-Cu-SiO_2

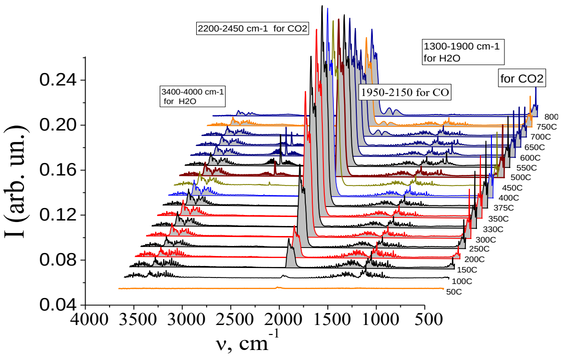

RF-Mg-SiO_2

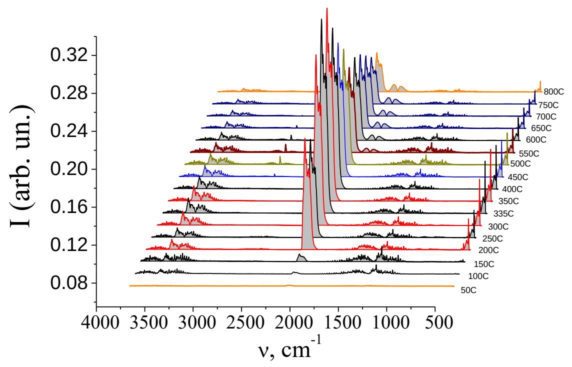

RF-Mn-SiO_2

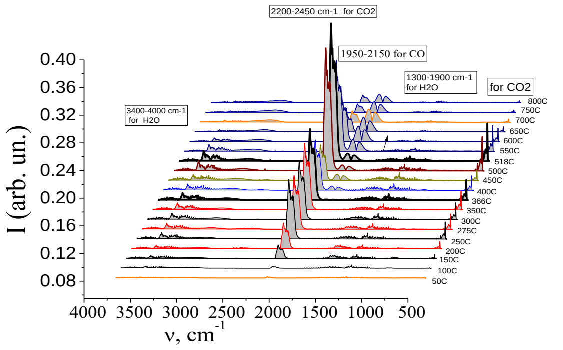

RF-Ni-SiO_2

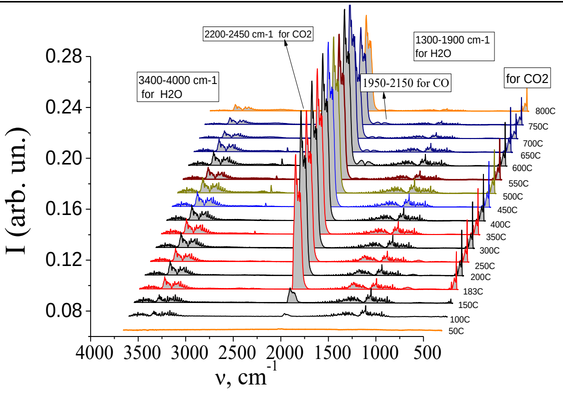

RF-Zn-SiO$_2$

Figure S1. FTIR spectrograms for the combustion of the samples.
